# Reducing the land use of EU pork production: where there’s swill, there’s a way

**DOI:** 10.1016/j.foodpol.2015.11.001

**Published:** 2016-01

**Authors:** Erasmus K.H.J. zu Ermgassen, Ben Phalan, Rhys E. Green, Andrew Balmford

**Affiliations:** a Conservation Science Group, Department of Zoology, University of Cambridge, Downing Street, Cambridge CB2 3EJ, UK; b RSPB Centre for Conservation Science, Royal Society for the Protection of Birds, The Lodge, Sandy SG19 2DL, UK

**Keywords:** Livestock, Sustainable animal diets, Feed, Food waste, Food security, Land use

## Abstract

Livestock production occupies approximately 75% of agricultural land, consumes 35% of the world’s grain, and produces 14.5% of anthropogenic greenhouse gas emissions. With demand for meat and dairy products forecast to increase 60% by 2050, there is a pressing need to reduce the footprint of livestock farming. Food wastes have a long history as a source of environmentally benign animal feed, but their inclusion in feed is currently banned in the EU because of disease control concerns. A number of East Asian states have in the last 20 years, however, introduced regulated, centralised systems for safely recycling food wastes into animal feed. This study quantifies the land use savings that could be realised by changing EU legislation to promote the use of food wastes as animal feed and reviews the policy, public, and industry barriers to the use of food waste as feed. Our results suggest that the application of existing technologies could reduce the land use of EU pork (20% of world production) by one fifth, potentially saving 1.8 million hectares of agricultural land. While swill presents a low-cost, low-impact animal feed, widespread adoption would require efforts to address consumer and farmer concerns over food safety and disease control.

## Highlights

• Food wastes are banned in animal feed in the EU due to disease control concerns.
• Many Asian states operate safe, centralised systems for recycling waste as feed.
• Asian-style food waste recycling could reduce the land use of EU pork by one-fifth.
• This would reduce environmental impacts without reducing pork quality or profits.
• Policy change will require pig industry, consumer, and political support.

## Introduction

Livestock production has a large and growing environmental impact. While providing one-third of all protein consumed by mankind (Herrero et al., 2009), livestock production occupies 75% of agricultural land (Foley et al., 2011), contributes 14.5% of anthropogenic greenhouse gas emissions (Gerber et al., 2013), and drives agricultural expansion in the tropics through the global trade in animal feed (Karstensen et al., 2013, Nepstad et al., 2006). With demand for meat and dairy products forecast to increase 60% by 2050 (Alexandratos and Bruinsma, 2012), there is growing recognition of the need to reduce the environmental impact of meat and dairy production.

Three principal strategies have been proposed to reduce the environmental impact of livestock: (1) reducing demand (Bajželj et al., 2014, Eisler et al., 2014, Fairlie, 2010; zu Ermgassen et al., 2014), principally in the developed world where meat and dairy consumption makes up a high proportion of food intake (Bonhommeau et al., 2013); (2) increasing efficiency, i.e. reducing the quantity of feed required per kg of meat or dairy produced (Garnett, 2013); and (3) changing animal diets to low-impact alternatives. Proposed novel, low-impact animal feeds include insects (Makkar et al., 2014), legumes (Jezierny et al., 2010), algae (Holman and Malau-Aduli, 2013), and bacteria (Byrne, 2014).

Low-impact animal feeds need not, however, be novel. Food waste has historically been recycled as livestock feed, particularly for pigs – cooked food waste fed to pigs is colloquially known as “swill”. Pigs are a monogastric species whose digestive system is well adapted for the conversion of food waste into animal protein (Westendorf, 2000a); food waste produced in early human settlements is thought to have attracted wild pigs, leading to their domestication around 10,000 years ago (Fairlie, 2010). Swill can be a high-quality animal feed that requires no additional land to be brought into production, and hence has minimal or even positive environmental impact (food waste otherwise posing a disposal challenge). However, the use of swill is controversial in some countries and there is marked geographic variation in its acceptance and regulation. Though the recycling of food waste as swill is actively promoted in many nations, including South Korea, Japan, Taiwan, and Thailand (Menikpura et al., 2013), it was banned in the European Union (EU) in 2002 after the UK foot-and-mouth disease epidemic, which is thought to have been started by the illegal feeding of uncooked food waste to pigs. Proponents claim that swill is a cheap, environmentally benign animal feed (Fairlie, 2010, Stuart, 2009, Wadhwa and Bakshi, 2013), but critics claim that it is unsafe and produces pork of poor quality (Garcia et al., 2005, House of Lords, 2014).

In this paper we address some of the controversies surrounding the recycling of food waste as animal feed and quantify the potential for food waste to replace conventional animal feed and reduce the environmental impact of meat production. First, we provide an overview of the history and regulation of swill feeding, focusing on the contrasting approaches taken by the EU and two East Asian states: Japan and South Korea. Second, we consider the role that swill can play in reducing the land required for meat production, through a quantitative case-study of pork production in the EU. We then discuss the impact of swill on other environmental impacts, including greenhouse gas emissions, before reviewing the barriers to swill feeding in Europe. We focus on the potential concerns of pig producers, the public, and policy makers. To finish, we briefly discuss the legal status of swill in other parts of the world, focussing on the world’s two largest pork producers: the USA and China.

## Swill in the EU, Japan, and South Korea

Although it is the archetypal pig feed, swill has been in and out of fashion in Europe. Swill was the prevalent pig feed in the early 20th century and was actively promoted by the UK government during the Second World War as a means of attaining food security (Fairlie, 2010). The popularity of swill feeding decreased in the late 20th century as the availability of abundant cheap grains led the pig industry to focus on increasing production efficiencies through grain- and soybean-based diets. The risks of uncooked swill were demonstrated in 2001 when a UK farmer illegally fed uncooked food waste to pigs, precipitating the 2001 foot-and-mouth disease outbreak, which cost the UK economy £8 billion (UK House of Commons report, 2002). In response, swill feeding was banned in the UK in 2001, with the ban extended across the EU the following year (EC, 2002). The ban still permits the feeding of some food wastes where it can be demonstrated that there is no risk of contamination with meat products, but this represents only a small proportion of all EU food waste (see Appendix A for further details of EU regulation and food waste recycling).

Today, the EU produces more than 20% of world pork, 34 kg of pork meat/person/year (FAO, 2014a), and relies on grain- and soybean-based feed, which has a sizeable environmental footprint. A life cycle assessment (LCA) of European pork production found that pork production causes €1.9 of damage to the environment (from eutrophication, acidification, land use, and greenhouse gas emissions) per kg of pork produced – in comparison, it costs the farmer on average €1.4 to produce each kg of pork (Nguyen et al., 2012). Most (75.4%) of this environmental burden stems from feed production – in particular, the farming of soybean meal. The expansion of soybean farming in South America to meet international demand for animal feed poses a significant threat to biodiversity and is a large source of carbon emissions from deforestation (Godar et al., 2015, Karstensen et al., 2013, Nepstad et al., 2006, Richards et al., 2014).

**Fig. 1:**
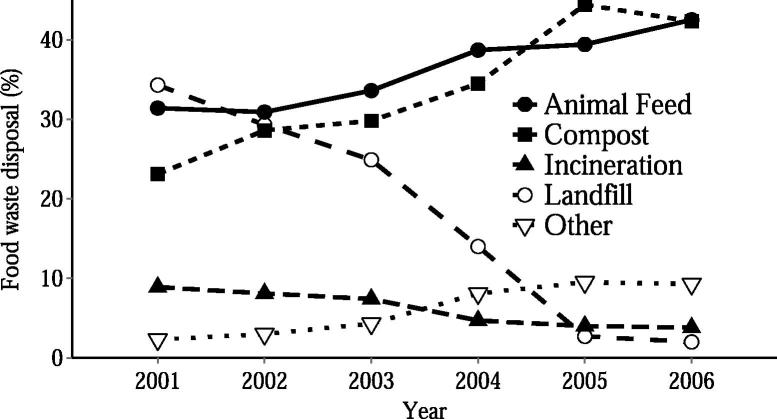
The end-uses of food waste in South Korea 2001–06, the most recent available data (Kim and Kim, 2010). After the introduction of food waste recycling legislation in 1997, South Korea achieved substantial increases in food waste recycling. The recycling of food waste for animal feed is shown as a solid line.

Not all modern pig production is reliant on grain and soybean feed. In the same year that the UK banned swill, the Japanese government introduced the opposite policy, promoting the inclusion of food waste in animal feed (Takata et al., 2012). South Korea and Taiwan have introduced similar food waste recycling systems (in 1997 and 2003, respectively). While the feeding of uncooked meat wastes to pigs can transmit diseases including foot-and-mouth and classical swine fever, appropriate heat treatment deactivates these viruses and renders food waste safe for animal feed (Edwards, 2000, Garcia et al., 2005, OIE, 2009). In these countries, the industry is tightly regulated: the heat treatment of food waste is carried out by registered “Ecofeed” manufacturers (see Appendix B for details of food waste recycling practices in Japan and South Korea). Where Japan and South Korea formerly sent substantial quantities of food waste to landfill, in 2006–07 they respectively recycled 35.9% and 42.5% of food waste as animal feed (Fig. 1) (Kim and Kim, 2010, MAFF, 2012a, MAFF, 2011).

## The potential for swill to reduce the land use of EU pork

To estimate the potential land use saving of a change in EU regulation to promote the recycling of food waste as animal feed, we performed three complementary analyses. (a) We estimated the current land use of EU pork production; (b) we used data from feed trials comparing food waste and conventional diets to determine how the incorporation of food waste in pig diets affects the amount of feed and land required for pig production; and (c) we estimated the availability of food waste suitable for pig feed in the EU. We then combined these results to estimate the potential impact of promoting swill on the land use of EU pork production.

In this analysis we use land use as a footprint metric to assess the potential environmental benefits of the re-legalisation and promotion of swill in the EU. While measuring land use alone does not capture all of the environmental impacts of meat production, we consider land use an informative (though incomplete) metric for this analysis because (a) land use represents the majority (55%) of the environmental costs of European pork production (Nguyen et al., 2012); and (b) land use is a valuable indicator of the biodiversity impacts of products (Mattila et al., 2011). While other biodiversity metrics have been used in life cycle assessment (LCA), there remains no consensus on their relative validity (Souza et al., 2015).

### The land use of EU pork production

**Fig. A3:**
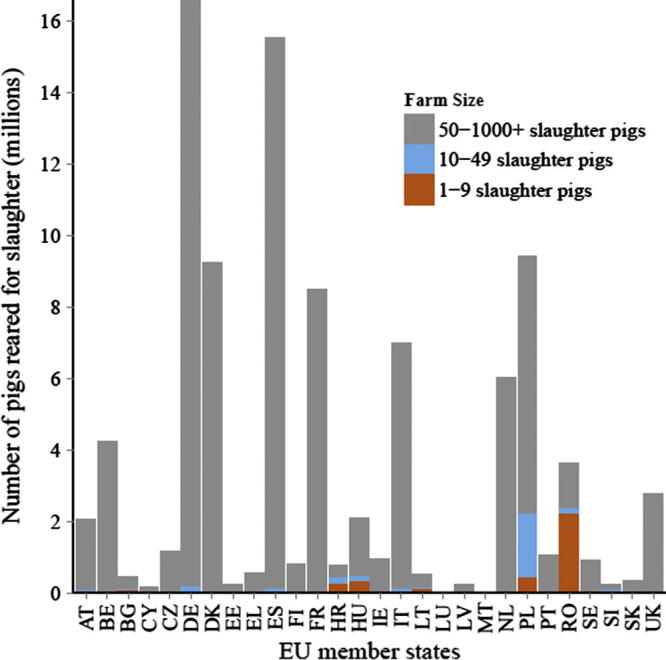
The number of slaughter pigs (all pigs reared for slaughter, excluding breeding animals and piglets <20 kg) reared on farms with different herd sizes in the EU in 2010. DE = Germany, ES = Spain, DK = Denmark, FR = France, PL = Poland, other country codes listed in electronic supplementary material, Table A3. Source: (Eurostat database, 2014).

To estimate the land use of EU pork, we calculated the land required across the entire lifecycle of pork production (breeding sows, piglets, and young and mature slaughter pigs) to grow the feed necessary to produce the 21.5 million tonnes of pork (live weight) which is produced in modern, large-scale pig production systems in the EU each year (for more details see Appendix C). The calculation was based upon weighted mean values of EU production statistics (e.g. the number of piglets weaned per sow per year, piglet mortality rates) and representative diets from the five leading producers of pork in the EU: Germany, Spain, Denmark, France, and Poland. These member states together represent >64% of EU pork production (Appendix C, Fig. A3).

We found relatively little variation in the estimated land use across all five sets of diets (4.02 m^2^/kg pork; range: 3.6–4.3 m^2^/kg) and determined that the land required to grow feed for EU pork was ca. 8.5 million ha (±0.7 Mha s.d.). Soybean production in 2010 represented ca. 15% of the total land area required for EU pig feed production, an area of 1.2 million ha (±0.2 Mha s.d.).

### The effect of swill on land required for pig production

**Fig. 2:**
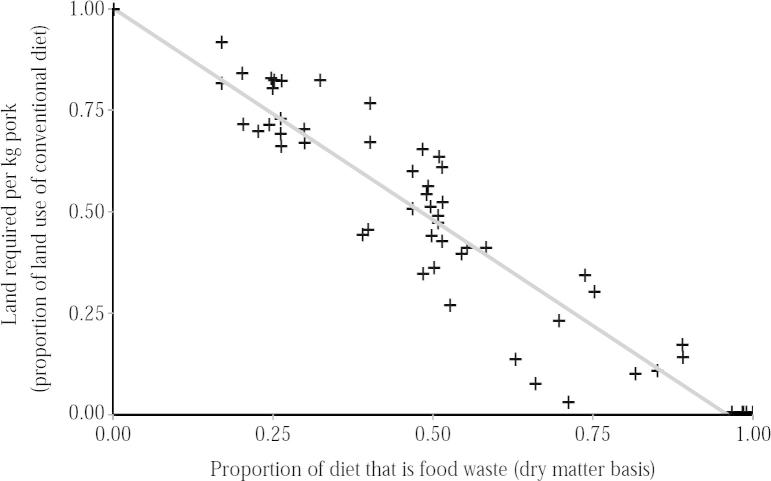
The inclusion of food waste in pig diets linearly reduces the land required per kg of pork live weight; *r* = 0.97, *n* = 78, *p* < 0.0001. This linear relationship reflects that the inclusion of food waste in pig feed (a) has no effect on the feed conversion efficiency (it substitutes conventional feed almost 1:1 on a dry matter basis (*t* = 1.15, *p* = 0.26)), and (b) does not have a large effect on growth rates (for more details see Appendix D). Some diets have a land use of zero, without being 100% swill: they contain a small amount of other ingredients, such as vitamins and minerals, which also do not require agricultural land.

To determine how the inclusion of food waste in pig feed influences the land required for pork production, we conducted a comprehensive literature review (Appendix D) to identify 18 feed trials comparing the growth performance of pigs on 23 conventional and 55 food waste-based diets. For each diet, we recorded the proportion of the diet that was food waste (on a dry matter basis) and calculated the land use per kg of pork (Appendix D). We found a strong linear relationship between the land use per kg of pork and the proportion of the diet made up by food waste (*r* = 0.97, *n* = 78, *P* < 0.0001; Fig. 2).

### The availability of food wastes in the EU

An estimated 102.5 million tonnes of food were wasted in the EU in 2015 (202 kg per person) (EC, 2010), from four principal waste streams: households (42%), manufacturing (39%), the food service/catering industry (14%), and retail (5% of food waste). These waste streams span the food supply chain, and so our definition of food wastes includes so-called “food losses” (food wasted during the post-harvest and processing stages; Parfitt et al., 2010), but excludes co-products (Appendix D) and agricultural wastes. The estimates of food waste are uncertain because of differing food waste definitions used by member states (e.g. classifications of green wastes), but are the best available data. We believe these figures are conservative estimates of EU food waste because they do not include agricultural wastes, which make up ca. 34% of all European food waste (Kummu et al., 2012), and we therefore used them as lower-bound estimates of the availability of food waste for use as pig feed in the EU.

Before estimating the quantities of food waste available for swill feeding, we made three adjustments. First, we subtracted the 3 million tonnes of manufacturing food waste (known in the processing industry as former foodstuffs) that are currently included in livestock feed in the EU (EFFPA, 2014). It is not clear whether these are excluded from the EU food waste data, so subtracting them makes our estimates of food waste available for pig feed conservative. Second, we allow for the fact that not all food waste defined by these statistics is available or suitable for pig feed. Only 35.9% and 42.5% of food waste is converted to animal feed in Japan and South Korea, respectively (Kim and Kim, 2010, MAFF, 2012a, MAFF, 2011). We assumed that a similar proportion can be used for the EU and took the mean of these two values (39.2%) to be the proportion of food waste available for recycling into animal feed, if swill feeding were legalised in the EU. Third, in the analyses above (Section ‘The effect of swill on land required for pig production’) we calculated the proportion of animal feed that is food waste on a dry matter basis. To calculate the proportion of EU pig feed that could be replaced by swill we therefore converted our waste estimates into tonnes of dry matter (Appendix E).

Finally, for comparison with the proposed EU swill-feeding scenario, we also calculated the potential for increasing the use of legal food wastes as animal feed under the current legislation. In this scenario, we estimated the land use savings of including in animal feed an estimated 2 million further tonnes of manufacturing food waste which are not currently used for animal feed but which could legally be fed to livestock (EFFPA, 2014).

### The potential for swill in the EU

We then used the results from Sections ‘The land use of EU pork production’, ‘The effect of swill on land required for pig production’ and ‘The availability of food wastes in the EU’ to estimate the potential for swill to reduce the land use of EU pork production (Appendix F). Our results indicate that if swill feeding were legalised and food waste recycled into animal feed at rates similar to those in Japan and South Korea, the land requirement of EU pork production could shrink by 1.8 million ha (1.7–2.0 Mha; 95% CI), from 8.5 to 6.7 million ha. This represents a 21.5% (19.6–23.5%; 95% CI) reduction in the current land use of industrial EU pork production. In doing so, swill would also replace 8.8 million tonnes of human-edible grains currently fed to pigs (Appendix F) – equivalent to the annual cereal consumption of 70.3 million EU citizens (FAO, 2014a).

Under the current EU legislation, only a small increase in the quantity of food waste used in animal feed is possible. These legal food wastes could reduce land use by 1.2% (1.0–1.4% or 0.08–0.12 million ha; 95% CI). While this legislation stands, efforts to promote the inclusion of legal food waste in animal feed should be supported in order to realise these modest improvements in environmental impact; our results suggest, however, that far greater gains could be achieved by re-legalising and promoting the use of swill.

Use of swill might also help reduce the impact of EU pork production on global ecosystems. The inclusion of food waste in pig feed would reduce the area of soybean required by 268,000 ha (0.25–0.29 Mha; 95% CI) (Appendix F). In Brazil, the source of the majority (60%) of EU soybean (FAO, 2014a), soybean production is forecast to expand by 10.3 Mha by 2023 (MAPA, 2014). While Brazil is not the sole source of EU soybean meal, the potential for EU swill-feeding to reduce demand for up to 268,000 hectares of soybean production could mitigate ca. 2.6% of the forecast expansion of soybean, reducing pressure on high-biodiversity tropical biomes accordingly.

## Swill: beyond land use

**Fig. 3:**
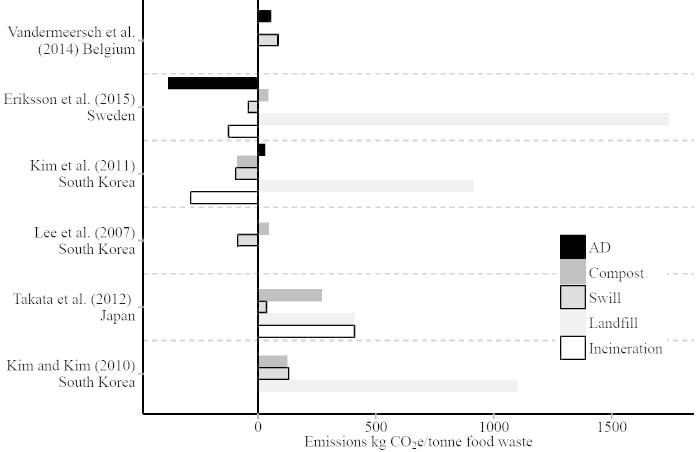
Results of six LCA studies reporting the greenhouse gas emissions per tonne of food waste for different disposal options, including recycling food waste as swill. Negative emissions mean that the process has a net negative carbon balance, ie the emissions avoided are larger than emissions released. Swill, for example, avoids emissions associated with the production of conventional feed, and anaerobic digestion (AD) avoids emissions from the fossil fuels it replaces. Where a study reported emissions for multiple food waste types, the mean emissions are shown, and none of the studies shown include land use change, a major source of agricultural emissions, when calculating the emissions avoided from swill feeding. The swill data for Vandermeersch et al. (2014) are for a 10% swill, 90% anaerobic digestion scenario. Gaps are left where studies did not report particular food waste disposal options, and the country of study is listed under each reference. Two further LCA studies (Ogino et al., 2012, Tufvesson et al., 2013) use different units (reporting results per kg of animal feed and per MJ of fuel energy, rather than per tonne of food waste) and so cannot be displayed for comparison. (Eriksson et al., 2015, Kim et al., 2011, Lee et al., 2007)

The substitution of conventional feed with food waste has the potential to reduce not only the land requirement for pork production, but also other environmental impacts associated with the production of animal feed, including greenhouse gas emissions and eutrophication The impacts of swill feeding on these other environmental effects are more difficult to estimate. For greenhouse gas emissions, while eight LCA studies have compared the recycling of food waste into animal feed with other food waste disposal practices (including incineration, anaerobic digestion, and composting), the calculated emissions vary substantially and are sensitive to local conditions and study assumptions (Fig. 3; Bernstad and la Cour Jansen, 2012). In particular, only one of these studies considers emissions associated with land use change, with the remaining studies therefore underestimating agricultural emissions of feed ingredients, such as soybean meal, by up to nine times (van Middelaar et al., 2013). Two multi-criterion LCAs have been conducted in the European context. Vandermeersch et al. (2014) compare two scenarios in Belgium: (1) sending retail food waste for anaerobic digestion and (2) recycling 10% as animal feed, with the rest sent for anaerobic digestion. This study found that the food waste feeding scenario scored better on 10 of 18 environmental criteria (including land use, marine eutrophication, and freshwater ecotoxicity), with anaerobic digestion scoring better on 8 criteria (including greenhouse gas emissions, ozone depletion, and freshwater eutrophication). Tufvesson et al. (2013) compare the use of manufacturing food wastes (such as bread wastes and fodder milk) for biofuel or animal feed in Sweden. They find that the use of these wastes as biofuel only results in environmental benefits (measured by greenhouse gas emissions, eutrophication, and acidification) if you do not take into account their potential use as animal feed. That is to say, they recommend the use of these wastes as animal feeds, instead using dedicated biofuel crops for biofuel (though this study did not take into account greenhouse gas emissions from indirect land use change resulting from the expansion of crop-based biofuels, nor the potential use of those biofuel crops as animal feed). As evidenced by the caveats above and the variable results presented in Fig. 3, the results of LCAs are often location, assumption, and study-dependent (Bernstad and la Cour Jansen, 2012). Future work should therefore analyse swill feeding and other uses of food waste in other EU member states, using alternative food waste sources, and taking into account all agricultural emissions.

## Barriers facing swill in the EU

While our EU-wide analysis is inevitably constrained by the available data, in particular by uncertainty about the quantity of food wastes produced in the EU and their nutritional content, we are confident that our principal conclusion is robust: a policy promoting the recycling of food waste as pig feed has substantial potential to reduce the global land use of EU pork production. When selecting animal feeds, however, there are many more considerations than simply their environmental impact. The adoption of swill feeding in the EU would require backing from pig producers, the public, and policy makers. We next consider the potential barriers from each interest group in turn.

### Support from pig producers

Pig producers want to produce pork of high quality, at affordable prices, with reliable profit margins, and the highest standards of food safety.

The 18 studies comparing food waste and conventional feed also reported a range of meat quality measures, allowing us to examine the effect of swill feeding on meat quality and palatability. We used linear mixed models to measure the effect of including food wastes in animal feed on 18 different measures of meat quality, which were each reported by three or more studies. Since pig age and breed, both important determinants of meat quality, varied among studies, study was included as a random effect. Further details of the methods are listed in Appendix G.

While swill does have more variable nutrient composition than conventional feeds (Westendorf, 2000b), swill feeding had little effect on meat quality, with no effect detected for 16/18 measures (Table 1). The two detected effects were weak and did not detrimentally affect pork quality or value. Pigs fed a 50% swill diet had 1.4% higher monounsaturated fats percentages (*t* = 3.39, data from 6 studies, *n* = 23, *p* = 0.017) and 13% greater meat marbling, the presence of streaks of fat within muscle tissue (*t* = 3.71, data from 6 studies, *n* = 22, *p* = 0.014). Pork marbling is known to increase the flavour and tenderness of pork (Brewer et al., 2001). Indeed, three studies intentionally fed food waste diets with a low lysine content in order to increase meat marbling (Witte et al., 2000). Removing these three studies from the analysis abolished the effect (*t* = −1.24, data from 3 studies, *n* = 10, *p* = 0.32). These results suggest that the inclusion of food wastes in animal diets can produce pork of similar quality to conventional diets, which may allay farmer concerns over product quality.

Farmers are also acutely concerned about the profitability of pork production. Feed makes up 55–72% of the costs of EU pig production and is subject to significant price volatility, with prices of conventional feed rising 70% from 2005–2012 (from $267 to $456/tonne) (AHDB Market Intelligence, 2013, AHDB Market Intelligence, 2006). Low-cost swill might therefore be a welcome alternative to conventional grain-based feed. Our results show that while swill feeding had no effect on feed conversion efficiencies (*t* = 1.15, *p* = 0.26), swill feeding did tend to slow pig growth rates (*t* = −4.71, *p* < 0.0001), which would necessarily increase labour and housing costs proportional to the number of extra days required to bring animals to slaughter. The relative merit of cheap, slower-growth swill and expensive, faster-growth conventional feed can be explored with a stylised example.

**Fig. 4:**
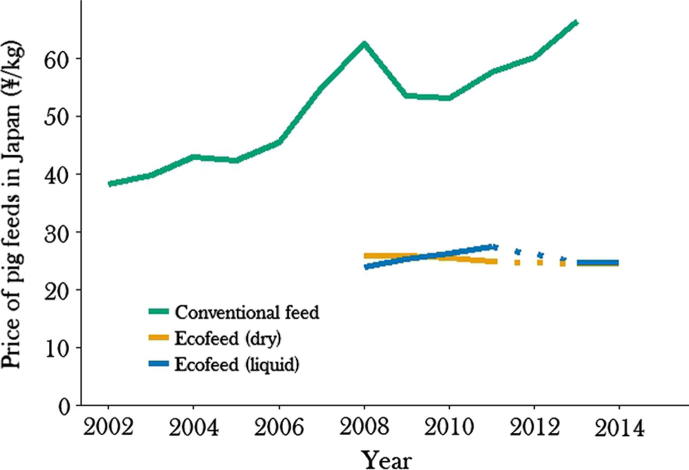
Prices of conventional pig feed and swill (Ecofeed) in Japan. Dry Ecofeed is fed as a dehydrated pellet, liquid Ecofeed is fed as a wet feed. Dotted lines are an interpolation between the 2011 and 2013 values. Data from: (MAFF, 2014, MAFF, 2013, MAFF, 2012b, MAFF, 2011, MAFF, 2010, MAFF, 2009).

Assume an EU pig farmer is considering converting to a 50% swill-diet. For simplicity, their current cost of production is €1/kg pork (the EU mean is approximately €1.4/kg pork (Nguyen et al., 2012)), of which 60% are feed costs (EU range of 55–72%), i.e. €0.6/kg. Our results suggest that a diet containing 50% food waste produces 13% lower growth rates, and so the farmer’s swill-fed pigs will need 13% longer to reach slaughter weight, making their conventional feed costs equal to €0.34/kg pork (1.13 ∗ 0.3, where 0.3 = the 0.6 of costs due to conventional feed ∗ 0.5, with the other half of the feed being swill). To conservatively estimate the cost savings of swill, we assume that all other costs also increase in proportion to the extra days required to reach slaughter weight (although fixed costs, such as depreciation and financial costs, make up 15–30% of the cost of production (AHDB Market Intelligence, 2013)). The farmer’s non-feed costs would therefore be 1.13 ∗ 40% = €0.45/kg pork. In this case, the farmer will have an overall lower cost of production if swill costs less than 70% the price of conventional feed (calculated as 1 – the cost of swill production/the cost of the equivalent conventional feed ∗ 100: [€1 − €0.34 − €0.45]/€0.3 ∗ 100). In the centralised food waste recycling systems, swill typically costs only 40–60% of conventional feed (20 vs. 50¥/kg in (Takahashi et al., 2012) and 167 vs. 278₩/kg in (Nam et al., 2000) and main text, Fig. 4). For this farmer, swill feeding would therefore improve profitability. Swill has a more variable nutritional content than conventional feeds (Westendorf, 2000b) and will not suit the business models of all farms, but it could help many to improve profitability. This is especially the case if swill-fed pork is marketed as a premium, low environmental impact product, as it is in Japan (“Eco-pork”, see Appendix B). There it receives an associated price-premium, which further boosts farm profits.

While swill feeding could benefit the bottom line of many individual farmers, there is concern that if the legalisation increased the risk of an outbreak of disease, such as foot and mouth or classical swine fever, the overall cost to the industry of such an outbreak could outweigh the financial gains (House of Lords, 2014). This concern is understandable given the £8 billion cost of the UK 2001 foot and mouth outbreak (UK House of Commons report, 2002). It is challenging to quantify the relative risk of a disease outbreak occurring under either of our two different policy scenarios: the status quo ban on swill and the centralised, regulated use of swill, and it is not certain which policy is lower risk. While it may be argued that a total ban on swill feeding is safer than the regulated use of swill, this ignores the illegal feeding of food waste on smallholder farms which occurs under current, “low-risk” legislation. A survey of 313 smallholder farms in the UK, for example, found that 24% of smallholders fed uncooked household food waste to their pigs (Gillespie et al., 2015). A process for the heat treatment and legal use of food wastes may improve on the current uncontrolled situation. It is worth noting that there have been no disease outbreaks linked to the use of swill in Japan and South Korea (Muroga et al., 2012, Park et al., 2013) and that the use of food waste as animal feed has consistently grown in both countries (by 125% in Japan from 2003–2013, Fig. A1 in Appendix B, and by 35% in South Korea from 2001–06, Fig. 1), suggesting strong farmer buy-in.

Finally, food safety precautions should include not only heat treatment but also checks for potential contaminants in food waste. Garcia et al. (2005) performed microbiological and chemical analysis of different Spanish food waste sources and found high levels of heavy metals and dioxins in some household and restaurant wastes. All other food wastes (e.g. retail meat, fruit, vegetable, and fish wastes) were deemed suitable for animal feed. The suspected sources of heavy metals were metal cans and piping. Contamination from these sources could be reduced through better collection, waste sorting, and storage procedures, as required by regulation in East Asian states (Appendix B).

### Support from the public

Our results and the East Asian case studies demonstrate that food waste can be safely recycled as pig feed to produce pork of high quality and low environmental cost. Despite this, swill has previously faced resistance because of concerns over consumer acceptability. For example, the co-operative, a UK food retailer, banned pork reared on food waste from shops in 1996 citing it “was not a natural feeding practice” (Stuart, 2009). This is an issue of public awareness, however, not food safety. Pigs were domesticated on a diet of swill, and as such, it could be argued that swill is no less “natural” than the practice of feeding vegetarian diets to omnivorous pigs in modern, industrial systems. Our review included a number of blinded trials finding no difference between the flavour (*n* = 4), colour (*n* = 7 for fat; *n* = 9 for meat), fat composition (*n* = 6), or overall palatability (*n* = 4) of conventional- vs. swill-fed pork (Table 1), suggesting that without labelling, consumers would not notice a difference. In fact, improving consumer awareness of swill has had positive effects in Japan, where certification has been introduced. A survey of consumers there found that those most knowledgeable about the pig industry showed the strongest approval of recycling food waste as feed (Sasaki et al., 2011). Public education may be beneficial in promoting the acceptance of swill in the EU.

### Support for policy change

Although currently illegal, there is some precedent for reappraising the legal status of swill. First, there is a legal mandate for improved food waste recycling under the EU Waste Framework Directive 2008/98/EC (EC, 2008), and second, similar animal feed regulation is being reconsidered in light of the EU’s deficit in protein sources for animal feed (EC, 2013).

**Fig. 5:**
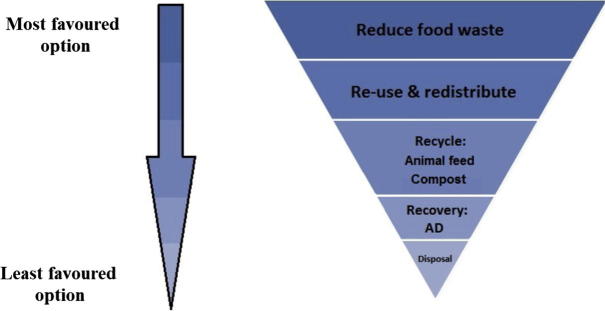
EU food waste hierarchy showing the different levels of waste disposal established under the EU Waste Framework Directive (EC, 2008). Recycling food waste as animal feed is preferable to composting, anaerobic digestion (AD), or disposal in landfill, the latter of which is to be phased out by 2025 under new legislative proposals (EC, 2014). The diversion of food waste for animal feed would not necessarily reduce the availability of inputs for the AD or composting industries, because the inevitable end product of the use of food waste as pig feed – pig manure – is itself highly suitable for both composting and anaerobic digestion (Bernal et al., 2009, Fairlie, 2010, Holm-Nielsen et al., 2009, Stuart, 2009). Image adapted from (Papargyropoulou et al., 2014).

The EU Waste Framework Directive stipulates that EU member states apply a waste management hierarchy to select disposal options in order of their environmental impact (Fig. 5). Under this legislation, the preferred options are to avoid food waste altogether or redistribute it to people. Next, the use of food waste as animal feed is preferable to composting, anaerobic digestion, or disposal in landfill (Papargyropoulou et al., 2014), though the legislation is notably not applied in this respect.

In 2001, the EU banned the use of all processed animal proteins (including pig by-products, such as tendons and trotters, which are fit for human consumption but not eaten by people for cultural or aesthetic reasons) in animal feed, in response to the Bovine Spongiform Encephalopathy crisis (EC, 2001). There are, however, no recorded cases of pigs, poultry, or fish ever naturally developing or transmitting diseases such as BSE (Andreoletti et al., 2007). After a scientific consultation (Andreoletti et al., 2007) and pressure from the animal feed industry (EFPRA, 2011, Searby, 2014), in 2013 the EU re-legalised the use of non-ruminant processed animal proteins in fish farming, and are currently considering its re-legalisation for use in pig and poultry feed (EC, 2013). It is plausible that swill could undergo a similar process of re-legalisation. It is worth noting that the ban on processed animal proteins is still expected to prevent “intra-species recycling”, i.e. the feeding of poultry waste to chickens, or pork waste to pigs. As swill can, and has always, contained pork wastes, swill-feeding legislation in the EU would have to permit this practice, as in the East Asian states described.

## Food waste as animal feed: beyond pigs and beyond the EU

This study has focussed on the potential to reduce the land use of EU pork through recycling food waste as swill because of the current EU ban on swill, and because pigs are an omnivorous species with a long history of food waste recycling. Pigs are, however, not the only animal that can consume diets containing food waste. A number of studies have trialled food waste diets for poultry (Boushy et al., 2000, Ruttanavut et al., 2011), fish (Cheng et al., 2014), and ruminants (Angulo et al., 2012, Ishida et al., 2012, Summers et al., 1980), and the environmental gains of food waste feeding for these species represents an area for further work.

The results of this study are also relevant to other parts of the world. We consider briefly here the state of swill feeding in the two largest producers of pork: China and the United States of America (together 55.3% of world production (FAO, 2014a)). By 2001, swill feeding had been banned in more than 18 US states (Spinelli and Corso, 2000, Stuart, 2009), and across the USA swill-feeding has seen a similar historical trajectory as in the EU: the growth of modern industrialised production systems and availability of abundant grain feed led to a decline in the number of pigs fattened on swill from 130,000 in 1960 to less than 50,000 in 1994 (Westendorf, 2000b). By 2012, 95% of US food waste was sent to landfill (US Environmental Protection Agency, 2012). However, swill has recently received renewed interest in the USA. The US Food Waste Challenge, launched in 2013, aims to promote the recycling of food waste, including the use of food waste as animal feed (HLPE, 2014).

In China the use of swill has remained common, and is one of the six highest-volume food waste disposal options nationally (Hu et al., 2012). Swill plays a particularly important role in backyard pig production (30–40% of pigs), where its low cost contributes to smallholder profitability (McOrist et al., 2011). As the Chinese pig industry becomes increasingly industrialised, however, there is a risk that the use of swill may decline (Fairlie, 2010), increasing the environmental impact of pork production, unless systems are put in place to produce swill for industrial pig producers. Centralised food waste recycling may be facilitated by the concentration of many industrial pork producers around densely populated urban areas (Gerber et al., 2005), thereby lowering transport costs and facilitating urban food waste recycling.

## Conclusions

As the demand for livestock products grows over the next half-century, we must identify strategies to reduce the environmental footprint of current systems of meat production. One strategy is the promotion of low-impact animal diets. Food waste, when heat-treated appropriately, as in the centralised food waste recycling systems of Japan and South Korea, can be a safe, nutritious form of animal feed. In this study we quantified the potential for swill to reduce the land use of EU pork production. While swill feeding is not a substitute for efforts to reduce food waste, our results suggest that changing EU legislation to promote the use of food waste as swill could substantially reduce the land use impacts of EU pork production. These environmental benefits can be achieved while improving the profitability of many farming businesses and delivering high quality pork products. Similar benefits may be seen in other parts of the world, where swill feeding is currently uncommon or illegal.

## Funding statement

E.K.H.J.zE is funded by BBSRC grant BB/J014540/1. BP is funded by the Zukerman research fellowship at King’s College Cambridge.

## Figures and Tables

**Table A1:** Food waste recycling in Japan, from 2001 to 2009, given as the percentage of food waste recycled for all purposes, including the production of Ecofeed, compost, and anaerobic digestion. Retail figures are a mean of wholesaler and retailer food waste recycling rates. Household food waste is not recycled in Japan, but is in South Korea (Stuart, 2009). Modified from (MAFF, 2012b, Takata et al., 2012).

Food waste source	2001	2002	2003	2004	2005	2006	2007	2008	2009
Manufacturing (%)	50	60	65	65	76	76	77	93	93
Retail (%)	23.5	26.5	30	29	42	44	45	48	47
Catering and food service (%)	9	8	11	12	14	16	16	13	16

**Fig. A1:**
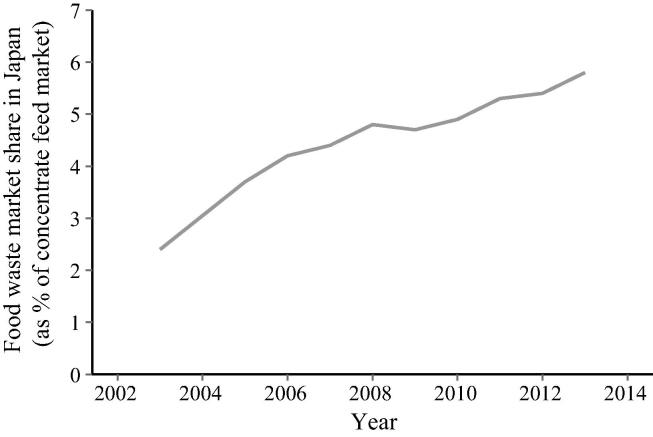
Food waste makes up an increasing proportion of animal feed in Japan. Source: (MAFF, 2014).

**Table A2:** Classification of food wastes under the Ecofeed certification scheme. To be certified, at least 5% of the entire feed must be “promoted” food wastes, with at least 20% of the total feed made up by a combination of both types of food waste. Adapted from Sugiura et al. (2009).

Type of food waste	Example
Ordinary	Distillery waste, beet pulp, rice bran, wheat bran, soybean dregs (excluding imported soybean dregs)
Promoted	Plate scraps, noodle debris, bread crumbs, cake crumbs, gluten debris, bean curd, mushroom-growing bed waste, sake lees, rice vinegar lees, tea dregs, squeezed fruit waste, coffee waste, cacao grounds, dairy plant wastes, frozen food plant wastes, cooking waste, waste oil, waste boxed lunches

**Fig. A2:**
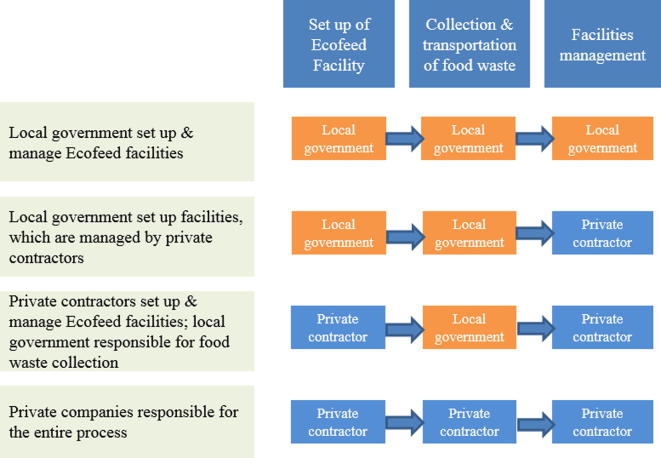
Ecofeed facilities in South Korea are managed through a combination of public and private partnerships.

**Table A6:** EU pork production statistic: summary of data listed in Tables A3, A5, and A7 in the electronic supplementary material. These statistics are based on a weighted mean of production statistics from 12 EU member states (representing >92.9% of EU industrial pork production) and representative diets from 5 EU member states (>64% of EU industrial pork production). Dietary ingredients do not sum to 100% because feed ingredients can belong to multiple groups (e.g. wheat bran is both a cereal and a co-product).

Summary statistics from Tables A3, A5, and A7	Value
*EU industrial pork production statistics*	
Weight at slaughter (kg)	114.8
Feed conversion ratio (kg feed/kg pig produced)	2.83
Piglets weaned/sow/year	25.1
Ratio sows:slaughter pigs	1:23.6

*EU industrial pork diet ingredients in percent (standard deviation)*	
Cereals (e.g. oats, corn, wheat)	71.4 (±8.5)
Soybean meal	9.5 (±1.6)
Agricultural co-products (excluding soybean meal, e.g. rapeseed meal, wheat bran, molasses)	14.8 (±5.3)
Vitamin and mineral supplements	3.0 (±1.0)
Other (e.g. peas, cassava, amino acid supplements)	5.0 (±6.0)

**Table A10:** Fresh weight and dry matter content of EU food wastes in 2015. The EU food waste figures (EC, 2010) assume that food waste was produced in the same proportions in 2015 as in 2006 (ie. households and retail wastes, for example, made up 42% and 5% of food wastes in 2015, as in 2006). The 39.2% figures (second row) represent the proportion of food wastes potentially recyclable as animal feed.

	Manufacturing food waste	Retail food waste	Catering food waste	Household food waste
EU food waste (tonnes)	38,786,404	5,122,616	14,343,324	43,029,974
39.2% of food waste recycled as feed (tonnes)^a^	15,204,271	2,008,065	5,622,583	16,867,750
Dry matter content (%) of food waste (95% CI)	29.8 (24.7–36.8)	23.3 (18.0–30.4)	21.5 (20.1–23.0)	26.0 (24.3–27.6)
Food waste recyclable as feed (DM tonnes)	4,530,873	467,879	1,208,855	4,385,615

a The figure for the percentage of manufacturing food wastes available for recycling excludes the 3 million tonnes of former foodstuffs which are already used for animal feed in the EU.

**Table A11:** Parameters used in land use calculations (Eq. (S2)).

Parameter	Value (95% confidence intervals)
${\mathrm{LU}}_{\mathrm{kg}\;\mathrm{pork}\;\mathrm{EU}}$	8.5 million ha
${\mathrm{coef}}_{\mathrm{FW}}$	−1.06
${\mathrm{FWprop}}_{\mathrm{household}}$	0.084 (0.079–0.089)
${\mathrm{FWprop}}_{\mathrm{manufacturing}}$	0.087 (0.070–0.104)
${\mathrm{FWprop}}_{\mathrm{retail}}$	0.009 (0.007–0.011)
${\mathrm{FWprop}}_{\mathrm{catering}}$	0.023 (0.022–0.025)
${\mathrm{FWprop}}_{\mathrm{currentlegislation}}$	0.011 (0.009–0.014)

**Table 1:** Relationships between the proportion of food waste in pig diets and measures of meat quality. P-values also shown for quadratic relationships, where suggested in the literature.

Meat quality (range or measurement units)	Number of studies (points)	Coefficient (SE)	*p*-Value	
Linear model	Quadratic model
Juiciness (0–1)	4 (13)	0.08 (0.04)	0.173	–
Marbling (1–10)	6 (22)	1.30 (0.35)	0.014	–
Dressing percentage (%)	12 (38)	0.89 (0.76)	0.264	–
Meat colour (1–5)	5 (17)	0.21 (0.28)	0.490	–
Meat lightness (*L*^∗^ value)	9 (33)	1.42 (0.81)	0.116	–
Meat redness (*a*^∗^ value)	9 (33)	−0.01 (0.27)	0.983	–
Meat yellowness (*b*^∗^ value)	9 (33)	0.32 (0.28)	0.283	–
Fat lightness (*L*^∗^ value)	7 (29)	0.99 (1.21)	0.443	–
Fat redness (*a*^∗^ value)	7 (29)	−0.08 (0.50)	0.872	–
Fat yellowness (*b*^∗^ value)	7 (29)	−0.39 (0.33)	0.282	–
Fat free lean percentage (%)	4 (15)	1.14 (0.89)	0.280	–
Flavour (0–1)	3 (7)	0.03 (0.02)	0.319	–
Overall palatability (0–1)	3 (7)	0.03 (0.05)	0.584	–
Monounsaturated fats (%)	6 (23)	2.83 (0.83)	0.017	–
Saturated fats (%)	6 (23)	−1.30 (1.01)	0.243	–
Polyunsaturated fats (%)	6 (23)	−1.50 (1.02)	0.186	–
6 (23)	−0.90 (0.55)	–	0.158
Backfat thickness (mm)	15 (53)	−0.58 (1.08)	0.599	–
15 (53)	−0.32 (0.60)	–	0.600
Drip loss (%)	3 (11)	−0.65 (1.33)	0.673	–
3 (11)	−0.32 (0.81)	–	0.729

## Appendix A – EU food waste legislation

EC regulation 1774/2002 regulation was introduced across the EU in 2002, after an initial ban on swill in the UK in 2001. It bans the use of catering wastes, whether from household, restaurant, or central kitchens for use as animal feed, effectively ending the 10,000 year-old practice of recycling food wastes as animal feed.

The continued use of food wastes is permitted only where it can be demonstrated that there is no risk of contamination with meat, fish, or other animal products. This requires either that a facility handle no animal products or they establish completely separate handling streams for animal and non-animal products, along with Hazard Analysis and Critical Control Point (HACCP) procedures. This prevents all but the largest businesses from recycling food waste as feed.

The proportion of food waste that is recycled as animal feed is therefore small. Of the 102.5 million tonnes of food waste produced in the EU per year (EC, 2010), it is estimated by the European Former Foodstuff Processors Association (EFFPA, the body which represents European processors of food wastes) only 3 million tonnes of manufacturing food wastes are currently recycled as animal feed, and that there are a further 2 million tonnes which could be legally recycled, under existing legislation (EFFPA, 2014). Food wastes recycled as animal feed are known in the processing industry as “former foodstuffs”.

The current method of disposal of most EU food wastes is not well known, because of limited data collection. The EU waste directive sets a target for 2025 that no biodegradable waste (including food wastes) be landfilled by 2025 (EC, 2014). Progress is slow, however, and large amounts of food waste are still disposed of in landfill. In parts of the UK, for example, food waste makes up to 48% of landfilled waste (House of Lords, 2014), which is associated with large greenhouse gas emissions (Fig. 3). Improved food waste recycling requires separate food waste collection, as occurred in 95% of Wales, 34% of Scotland, 26% of England, and 4% of Northern Ireland in 2013 (House of Lords, 2014).

## Appendix B – Japanese and South Korean food waste legislation

### Japanese food waste legislation

In 2001 Japan introduced the Promotion of Utilization of Recyclable Food Waste Act (Food Waste Recycling Law) which has seen large increases in food waste recycling, including the recycling of food wastes into animal feed (Table A1). Animal feed from recycled food waste is known as “Ecofeed”.

The Food Waste Recycling Law regulates the collection, transport and storage of food wastes and Ecofeed products. In 2007 the law was amended to make animal feed the priority use of food wastes, in preference to composting or incineration, and to create “recycling loops” by requiring companies which produce food waste to preferentially purchase Ecofeed-reared pork (Takata et al., 2012). In 2006, Japan successfully recycled 52.5% of its manufacturing, retail, and catering food waste as animal feed (MAFF, 2011) – the remaining portion being composted, incinerated, or landfilled on the grounds of being inedible, like orange peels or rotten food, or being produced in locations without the necessary recycling infrastructure. Recycling rates differ between industries: less food is currently recycled from catering outlets, which are diffusely distributed and individually have small waste streams (16% of food waste recycled in 2009, up from 9% in 2001) than from food manufacturing plants, which are more concentrated and produce larger waste streams (93%, up from 50% in 2001).

Ecofeed manufacturers (see http://ecofeed.lin.gr.jp/map.cgi) operate under Japanese food safety law which requires that food waste containing meats must be heated for a minimum of 30 min at 70 °C or 3 min at 80 °C (Sugiura et al., 2009). Household wastes (31.6% of all food waste) are not currently recycled into animal feed in Japan because they are vulnerable to contamination by foreign objects (e.g. cutlery (Sugiura et al., 2009)), although household wastes are recycled in South Korea (Stuart, 2009), where food waste is screened for potential contaminants before use. The use of meat wastes in ruminant (cattle, goat and sheep) diets is banned because of concerns about Bovine Spongiform Encephalopathy (BSE), a disease that does not affect pigs or poultry (Andreoletti et al., 2007).

Since its introduction, the Ecofeed market has grown year-on-year (Fig. A1), and food wastes made up 5.8% of all concentrate animal feed (for pigs, poultry, and ruminants) in 2013. To promote Ecofeed further, the government has provided financial support and introduced Ecofeed certification. Ecofeed receives support under the ¥23 billion ($194m) “Grant to Create a Strong Agricultural Industry” and the ¥89 million ($750,000) “Urgent Plan to Increase Ecofeed Production” (MAFF, 2014). Certification was introduced in March 2009. To be certified, animal feeds must contain more than 20% food waste (with at least 5% of the entire feed made up by “promoted food wastes”, which include noodle debris, plate scraps, waste oil, and coffee grounds; see Table 3). Forty-nine feeds were Ecofeed certified as of September 2013. Similarly, certification of products from livestock reared on Ecofeed was introduced in 2011, with 8 brands certified by September 2013).

### South Korean food waste regulation

The recycling of food waste in South Korea is regulated under both the Wastes Control Act (Ministry of Environment, 2010a) and the by the Control of Livestock and Fish Feed Act (Ministry of Agriculture, Food, and Rural Affairs, 2010). In 2006, 42.5% of all food waste was recycled as animal feed (the most recent data available; Kim and Kim, 2010).

Under the Control of Livestock and Fish Feed Act Article 8, food waste can only be included in animal feed if it has been treated at registered feed production facilities – of which there were 259 facilities in 2010 (Ministry of Environment, 2010b). Facilities which produce wet feed from food waste are often located on-farm to minimise transport costs, while facilities which produce dry feed are often near urban centres and can be operated by either local government or private firms (see Fig. A2 for information on the possible management structures; Ministry of Environment, 2012).

The process of swill production is standardised under Article 11 of the Control of Livestock and Fish Feed Act. In all cases, food waste must be heat treated for 30 min to a core temperature of at least 80 °C in order to be included in animal feed; the exact process differs between dry and wet feed. For the production of dry feed, food waste is typically dehydrated by mixing with air heated to 390 °C. This method sterilises the feed, increases the feed shelf life, and avoids destroying nutrients (National Institute of Environmental Research, 2012). Wet feed production typically involves two steps. First, the feed is sterilized by heating to more than 80 °C. Second, the moisture content of the feed is standardised to 70–80% by mixing with corn or rice husks. Both these production processes must also meet the conditions of the Article 14 of the Control of Livestock and Fish Feed Act, which sets limits on the acceptable standard of hazardous materials in animal feed, such as heavy metals and fungal toxins.

It is worth noting that foot-and-mouth disease outbreaks in Japan (2010) and South Korea (2010–11) were not linked to swill feeding practices (Muroga et al., 2012, Park et al., 2013).

## Appendix C – The land use of EU pork production

The great majority of EU pork production occurs in industrial, indoor systems, with 95% of all pork in 2010 coming from farms with more than 50 slaughter pigs (pigs >20 kg, reared for slaughter; Fig. A3). Pork from farms holding more than 50 slaughter pigs is hereafter named “industrial” production. While the diets of pigs in smallholder systems (<50 pigs per farm) may be more variable, industrial pork production is characterised by animals fed grain- and soybean-based diets, maximising feed efficiency, with animal feed sourced off farm, thus decoupling traditional livestock and crop nutrient cycling (Naylor, 2005). As this analysis is concerned with the potential for food waste to replace grain-based feed, we limited our calculation of the land use of EU pork to the 21.5 million tonnes of pork produced in EU industrial systems annually (Eurostat database, 2014). For reference, we list characteristic statistics for EU industrial pork production in Table 4.

The land required per kg of EU pork production, ${\mathrm{LU}}_{\mathrm{kg}\;\mathrm{pork}\;\mathrm{EU}}$, as calculated as the sum, across all dietary ingredients, *i*, and pig types, *t*, (sows, piglets, and young and mature slaughter pigs) of the land area required to grow the feed necessary to produce 1 kg of pork (live weight) (Eq. (S1)).

(S1) $${\mathrm{LU}}_{\mathrm{kg}\;\mathrm{pork}\;\mathrm{EU}}=\underset{t\text{,}i}{\sum }\left({\mathrm{FCE}}_{t}*{\mathrm{Prop}}_{t\text{,}i}*\frac{1}{{\mathrm{yield}}_{i}}*{\mathrm{EA}}_{i}\right)$$

*FCE* (Feed Conversion Efficiency) is the kg of feed required per kg of pork live weight gain; *Prop_i_* is the proportion of the diet that is ingredient, *i*, on a dry matter basis (Appendix E); $\frac{1}{{\mathrm{yield}}_{i}}$ is the area required to produce 1 kg of raw product (ha/kg raw product, e.g. soybeans). Finally, *EA_i_* is an economic allocation factor for the proportion of the land required to produce ingredient *i* which is allocated to ingredient *i*, rather than to other co-products. Allocation is used to divide land use between different co-products of a crop, and was weighted according to the economic value of co-products, as in previous life cycle analyses of livestock products (de Vries and de Boer, 2010, Guinée et al., 2004). Soybeans, for example, are processed into both soybean meal, a common pig feed ingredient, and soy oil; soybean meal makes 60% of soybean value, and soy oil the other 40%, and so soybean meal has an EA of 0.6 (see electronic supplementary material, Table A4).

*FCE* as calculated according to Nguyen et al. (2011) (detailed in electronic supplementary material, Table A5), using weighted mean values of national pig production statistics. Production statistics were weighted according to the proportion of EU production that occurred in each state in 2010, calculated using data available from 12 EU member states (Austria, Belgium, Czech Republic, Denmark, France, Germany, Ireland, Italy, the Netherlands, Spain, Sweden and the UK) (AHDB Market Intelligence, 2013). Member states for which production data were not available were assigned production statistics from states with similar pig production (e.g. the piglet mortality rate in Poland was estimated from Czech production figures; see electronic supplementary material, Table A3).

*Prop_i_* as calculated using representative diets from the five leading producers of industrial pork in the EU: Germany, Spain, and Denmark, France, and Poland, which together represent >64% of EU industrial pork production (diets listed in electronic supplementary material, Table A7). Dry matter contents were based on values from (FAO, 2014b) and ${\mathrm{Prop}}_{i}$ as calculated using the EU weighted mean, as above.

Yields were calculated from a five-year mean (2008–12) of production from the EU’s largest national producer in 2012. For example, France produced 40.3 million tonnes of wheat in 2012 (30% of EU production), with a five year mean yield of 7.1 tonnes/ha (FAO, 2014a). The yields of crops not grown in large quantities in the EU (soybeans, palm oil, tapioca, and safflower oil) were based on a five-year mean of the nation that exported the greatest quantity of that crop into the EU in 2010. To ensure land use was estimated conservatively, we assumed 25% of the Brazilian soybean harvest was double cropped, as in an LCA of Argentinian soybean production (Dalgaard et al., 2008). Our study is concerned with the land required to produce pig feed, and so the land occupied by pig farms themselves (a very small proportion of the area required for feed production) is not considered, as in previous LCAs of pig production (Dalgaard et al., 2008, Reckmann et al., 2013).

Finally, to calculate the total area required for EU pork production, ${\mathrm{LU}}_{\mathrm{kg}\;\mathrm{pork}\;\mathrm{EU}}$ was multiplied by the total output of EU industrial pork production, 21.5 million tonnes (live weight) (Eurostat database, 2014).

## Appendix D – Modelling the effect of food waste on land use of pork production

To determine the effect of food waste on the land required for pork production, we searched for relevant literature published between January 1900 and September 2014. To maximise the likelihood of finding relevant studies, we used multiple paired search terms, including ANIMAL FEED, FEED, LIVESTOCK, PIG, or PORK and WASTE, FOOD WASTE, FORMER FOODSTUFF, ECOFEED, SWILL, and RECYCLING. We read the references of identified studies and followed up any which appeared relevant. Studies were translated from the original Japanese or South Korean, where required. We applied the following inclusion criteria for our study: we included only studies which recorded the feed intake and the weight gain of pigs fed conventional and food waste diets, as well as the proportion of the diet that was made up of food wastes.

We identified 18 feed trials comparing the growth performance of pigs on 23 conventional and 55 food waste-based diets (Chae et al., 2003, Chae et al., 2000, Cho et al., 2004, Kjos et al., 1999, Kumar et al., 2014, Kwak and Kang, 2006, Maeda et al., 2014, Márquez and Ramos, 2007, Mitsumoto et al., 2006, Moon et al., 2004, Myer et al., 1999, Nam et al., 2000, Ohmori et al., 2007, Sirtori et al., 2010, Takahashi et al., 2013a, Takahashi et al., 2013b, Takahashi et al., 2012, Westendorf et al., 1998). Each feed trial evaluated how pig growth and meat quality were affected by the inclusion of food waste in pig feed; these feed trials mimicked conventional production systems where possible, using, for example, pig breeds common in modern production systems.

For each diet (listed in electronic supplementary material, Table A8), we recorded the proportion of each diet that was food waste (on a dry matter basis) and calculated the land requirement per kg of pork according to Eq. (S1), assigning food waste a land use of zero. The distinction between co-products and food waste can be a grey area. Potato peels or brewing wastes, for example, may be considered a food waste or co-product, depending on whether or not they are a traded commodity. In order to conservatively estimate the land use savings of swill feeding, we classify potato peels, brewing wastes, beet pulp, and dairy wastes (e.g. whey), which are not infrequently used for animal feed in the EU, as co-products and assign them a land requirement accordingly. Previous studies have shown that, compared with grain-based feed, the inclusion of co-products in animal feed can lower the environmental impact of meat production (Elferink et al., 2008), though soybean meal is a notable exception.

Having calculated the land use of each diet, we fitted a linear model to determine the effect of the inclusion of food waste on the land required per kg pork (Fig. 2). To allow comparison across different studies, which used different conventional diets (and therefore the land use of conventional diets differed between studies), we fit the land use of each diet as a proportion of the land use of the conventional pig feed in that study. We used untransformed proportion data in our model because errors were approximately normally distributed and applying the logit transformation (Warton and Hui, 2011) reduced model fit (*r* = 0.97 vs. 0.94). We tested for differences between four sources of food waste (household wastes, retail [e.g. supermarket] wastes, food service industry [catering or restaurant] wastes, or manufacturing [e.g. sandwich factory] wastes), because food waste composition can vary according to source (Esteban et al., 2007, Zhang et al., 2007), but found no difference between a model pooling food wastes and one differentiating them according to source (*F*_3,76_ = 1.78; *p* = 0.157). The linear model for pooled food wastes was therefore used for subsequent steps in the analysis. All statistical modelling was performed in R version 3.0.1 (R Core Team, 2013).

We find that the inclusion of food waste in pig diets linearly reduces the land required per kg of pork live weight (*r* = 0.97, *n* = 78, *p* < 0.0001). This linear relationship reflects that the inclusion of food waste in pig feed (a) has no effect the feed conversion efficiency (it substitutes conventional feed 1:1 on a dry matter basis), and (b) does not have a large effect on growth rates. While food waste diets do produce slower growth than conventional diets (*t* = −4.71, *p* < 0.0001), in part because their nutrient content is more variable, this effect is relatively small (see Section ‘Support for policy change’). If food waste diets did slow growth rates substantially, then the data would be poorly approximated by a linear relationship, and we would see many points in the upper right quadrant of Fig. 2 (ie. above the linear model fitted). To use an example, if a pig fed a 50% food waste diet grows much slower than a pig fed a conventional grain diet, then the animal’s total grain use, and the land required for that diet, would decrease by less than 50%, because the animal would be alive for longer before reaching slaughter weight, and would be eating some grain on each of those additional days. We find instead that the relationship is well described by a linear model (*r* = 0.97) and has a slope steeper than 1 (*t* = −59.2, *p* < 0.0001).

## Appendix E – EU food waste on a dry matter basis

Food waste diets differ greatly in their moisture content, depending on whether fed as a pellet or liquid. We therefore modelled the land required per kg of pork as a function of the proportion of animal feed that is food waste on a dry matter basis.

To determine the proportion of conventional feed that may be replaced by swill we therefore needed to first estimate the dry matter content of EU food wastes. We searched for studies reporting the dry matter content of food wastes by conducting a literature search for studies published between January 1900 and August 2014 using Thomson Reuter’s Web of Science® and Google Scholar in August 2014. To maximise the likelihood of capturing relevant studies, we used multiple paired search terms, including the same search terms as in Appendix D. We also searched using the search terms: FOOD WASTE and BIOGAS or ANAEROBIC DIGESTION because the dry matter of food waste is often reported in studies evaluating the potential use of food waste as a biofuel feedstock. We read the references of identified studies and followed up those which appeared relevant.

This literature review identified 220 estimates of the dry matter percentage of food wastes from all four food waste sources (a minimum of 50 estimates for each source, listed in electronic supplementary material, Table A9). We recorded the food waste source, region of origin (EU or non-EU), and dry matter percentages for each estimate. Studies of mixed municipal wastes were not included because of potential contamination with non-food items (e.g. paper and garden wastes) and when a range of dry matters for a particular food waste was quoted, the mean was used. The data were logit transformed (Warton and Hui, 2011) and explored using ANOVAs. There was no difference between the dry matter of food wastes sampled in EU and non-EU countries (*F*_1,215_ = 1.42, *p* = 0.235), so the dry matter estimates of food wastes from all regions were pooled. There was a significant difference between the mean dry matter contents of different food waste sources (Table 5; *F*_3,216_ = 2.90, *p* = 0.036) and bootstrapped 95% confidence intervals for the mean dry matter content of each food waste source were computed by resampling 10,000 times with replacement (Table 5).

## Appendix F – Land use saving of swill feeding in the EU

The potential land use saving of EU swill feeding was calculated according to Eq. (S2), where ${\mathrm{LU}}_{\mathrm{kg}\;\mathrm{pork}\;\mathrm{EU}}$ the total land area required to produce pork in the EU (main text, Section ‘The land use of EU pork production’, Appendix C, and Table A11), ${\mathrm{coef}}_{\mathrm{FW}}$ is the slope of the relationship between land use and the proportion of pig feed from food waste (main text, Section ‘The effect of swill on land required for pig production’, Fig. 1, and Table A11), and ${\mathrm{FWprop}}_{y}$ is the proportion of pig feed in the EU that could be replaced by different food waste sources, *y* (main text, Section ‘The availability of food wastes in the EU’ and Table A11). Confidence intervals (95%) for the land use savings were computed using the bootstrapped values of the dry matter content of EU food wastes (Table A10).

(S2) $$\text{Reduction in area required}\;(\text{ha})={\mathrm{LU}}_{\mathrm{kg}\;\mathrm{pork}\;\mathrm{EU}}*\underset{y}{\sum }(-{\mathrm{coef}}_{\mathrm{FW}}*{\mathrm{FWprop}}_{y})$$

This calculation is run twice, first to estimate the land use savings possible if EU legislation were changed and 39.2% of EU food waste were included in pig feed, and second to estimate the land use savings possible under the current legislation. In the latter case, we measure the land use saving possible if two million tons of legal food wastes (known as former foodstuffs), which are not currently used in animal feed, were included in pig feed (see main text, Section ‘The availability of food wastes in the EU’ and Table A11).

As well as calculating the total land use savings of swill feeding, we also report our results in terms of how the use of swill could reduce demand for both cereals (in tonnes) and soybean production (in hectares). Our previous calculations (Appendix D and E) show that swill can replace 20.3% of EU pig feed (on a dry matter basis). As 71.4% (±8.5% s.d.) of EU conventional pig feed (totalling 60.8 million tonnes) is comprised of cereals, including wheat, barley, oats, triticale, and corn, this suggests swill can replace 8.8 million tonnes of cereals currently used for pig feed. This quantity is equivalent to the annual cereal consumption of 70.3 million EU citizens (124.9 kg cereals/yr/capita of wheat, barley, corn, rye, and oats) (FAO, 2014a). Similarly, when calculating the area of soybean production potentially spared by swill feeding, the area calculated includes only the 9.5% (±1.6% s.d.) of our EU pig feed diets which is comprised of soybean. i.e. we do not double count the savings made from swill replacing soybean and cereals in conventional pig feed.

Eq. (S2) assumes that the food wastes used in the 38 identified food waste diets are similar in nutrient composition to EU food waste. We believe this to be a valid assumption because: (1) these diets include a representative range of food waste sources, from bakery wastes to household wastes, to supermarket leftovers; (2) we found no difference between the dry matter content of food wastes in EU and non-EU countries suggesting that food waste compositions, though variable between samples, do not differ systematically between locations; and (3) the high rates of food waste recycling as swill in countries such as Japan (35.9%) and South Korea (42.5% of food waste) suggest that many food wastes are suitable as pig feed, if the correct infrastructure is in place to treat them.

## Appendix G – The meat quality of pigs reared on food waste diets

We fitted linear mixed models for 18 meat quality measures which were reported by three or more of the identified studies (Appendix D and Table 1, main text). Since pig age and breed, both important determinants of meat quality, varied between studies, study was included as a random effect. Where studies in the literature have postulated a quadratic relationship between the proportion of food waste in diets and meat quality measures (Kjos et al., 1999), quadratic models were also tested. All mixed modelling used the “lme4” package in *R* and *p*-values for fixed effects were calculated using Kenward–Roger approximations generated using the “pbkrtest” package (Halekoh and Højsgaard, 2014), and the assumptions of statistical models were tested using a full residual analysis.

When comparing the flavour, juiciness, and overall palatability of pork reared on different diets, because different scales were used in different studies, scores were standardised as a proportion of the maximum potential score. Marbling scores were standardised to a 1–10 scale, in accordance with the National Pork Producers Council scoring system (Takahashi et al., 2012). Colour data were similarly standardised to a 1–5 scale for inter-study comparison. Where drip loss was recorded after multiple time points, the latest recording was used to maximise the likelihood of detecting a difference between the pork reared on conventional and food waste diets.

## H Supplementary material

Supplementary Tables A3–A5 and A7–A9 Supplementary material contains Tables A3–A5 and A7–A9.
